# Supplementary material for: Dietary inclusion of nitrite-containing frankfurter exacerbates colorectal cancer pathology and alters metabolism in APCmin mice
Source: NPJ Sci Food. 2022 Dec 28;6:60. doi: 10.1038/s41538-022-00174-y (PMC9797476; doi:10.1038/s41538-022-00174-y)
Supplement: Supplementary file 2 — Reporting Summary [file 41538_2022_174_MOESM2_ESM.pdf]

nature portfolio

Corresponding author(s): Brian Green

Last updated by author(s): 2022-25-10

Reporting Summary

Nature Portfolio wishes to improve the reproducibility of the work that we publish. This form provides structure for consistency and transparency in reporting. For further information on Nature Portfolio policies, see our Editorial Policies and the Editorial Policy Checklist.

Statistics

For all statistical analyses, confirm that the following items are present in the figure legend, table legend, main text, or Methods section.

n/a	Confirmed	
☐	☒	The exact sample size (*n*) for each experimental group/condition, given as a discrete number and unit of measurement
☐	☒	A statement on whether measurements were taken from distinct samples or whether the same sample was measured repeatedly
☐	☒	The statistical test(s) used AND whether they are one- or two-sided<br>*Only common tests should be described solely by name; describe more complex techniques in the Methods section.*
☐	☒	A description of all covariates tested
☐	☒	A description of any assumptions or corrections, such as tests of normality and adjustment for multiple comparisons
☐	☒	A full description of the statistical parameters including central tendency (e.g. means) or other basic estimates (e.g. regression coefficient) AND variation (e.g. standard deviation) or associated estimates of uncertainty (e.g. confidence intervals)
☐	☒	For null hypothesis testing, the test statistic (e.g. *F*, *t*, *r*) with confidence intervals, effect sizes, degrees of freedom and *P* value noted<br>*Give P values as exact values whenever suitable.*
☒	☐	For Bayesian analysis, information on the choice of priors and Markov chain Monte Carlo settings
☒	☐	For hierarchical and complex designs, identification of the appropriate level for tests and full reporting of outcomes
☐	☒	Estimates of effect sizes (e.g. Cohen's *d*, Pearson's *r*), indicating how they were calculated

Our web collection on statistics for biologists contains articles on many of the points above.

Software and code

Policy information about availability of computer code

Data collection	N/A
Data analysis	N/A

For manuscripts utilizing custom algorithms or software that are central to the research but not yet described in published literature, software must be made available to editors and reviewers. We strongly encourage code deposition in a community repository (e.g. GitHub). See the Nature Portfolio guidelines for submitting code & software for further information.

Data

Policy information about availability of data

All manuscripts must include a data availability statement. This statement should provide the following information, where applicable:

- Accession codes, unique identifiers, or web links for publicly available datasets
- A description of any restrictions on data availability
- For clinical datasets or third party data, please ensure that the statement adheres to our policy

Provide your data availability statement here.

Human research participants

Policy information about studies involving human research participants and Sex and Gender in Research.

Reporting on sex and gender	NOT APPLICABLE
Population characteristics	NOT APPLICABLE
Recruitment	NOT APPLICABLE
Ethics oversight	NOT APPLICABLE

Note that full information on the approval of the study protocol must also be provided in the manuscript.

Field-specific reporting

Please select the one below that is the best fit for your research. If you are not sure, read the appropriate sections before making your selection.

☒ Life sciences ☐ Behavioural & social sciences ☐ Ecological, evolutionary & environmental sciences

For a reference copy of the document with all sections, see nature.com/documents/nr-reporting-summary-flat.pdf

Life sciences study design

All studies must disclose on these points even when the disclosure is negative.

Sample size	Groups were composed of: control (n=9; standard chow), or chow with a 15% incorporation of pork mince (n=9), pork sausage (n=10), or nitrite-containing frankfurter sausage (n=10).
Data exclusions	Data were not excluded.
Replication	In vivo studies involved one replication but were longitudinal and where possible (e.g. blood/urine biomarkers, bodyweight etc.) repeated measures were taken at different time points.
Randomization	Mice were allocation to dietary groups at random. Tissue sections, blood, urine and feacal samples were analyses in a randomised order. tumour counting, histology and biochemicals
Blinding	Tumor counting and histology were conducted in a blinded manner and then unblinded for analysis. Metagenomic sequencing and mass spectrometry analysis took place in a blinded manner and were unblinded after quantitifications. Mouse groups and diets were not blinded.

Reporting for specific materials, systems and methods

We require information from authors about some types of materials, experimental systems and methods used in many studies. Here, indicate whether each material, system or method listed is relevant to your study. If you are not sure if a list item applies to your research, read the appropriate section before selecting a response.

Materials & experimental systems

n/a	Involved in the study	
☐	☒	Antibodies
☒	☐	Eukaryotic cell lines
☒	☐	Palaeontology and archaeology
☐	☒	Animals and other organisms
☒	☐	Clinical data
☒	☐	Dual use research of concern

Methods

n/a	Involved in the study	
☒	☐	ChIP-seq
☒	☐	Flow cytometry
☒	☐	MRI-based neuroimaging

Antibodies

Antibodies used	No individually purchased antibodies were used apart from a proprietary antibody within the malondialdehyde (MDA) ELISA kit supplied by Abcam, Cambridge, UK. The details of this antibody are not available.
Validation	Not applicable.

Animals and other research organisms

Policy information about studies involving animals; ARRIVE guidelines recommended for reporting animal research, and Sex and Gender in Research

Laboratory animals	C57BL/6J-ApcMin
Wild animals	Not applicable.
Reporting on sex	All mice used were female to eliminate gender as a potential confounding factor.
Field-collected samples	Not applicable.
Ethics oversight	Studies in mice underwent Ethical Review and were performed in accordance with the UK Animals (Scientific Procedures) Act 1986.

Note that full information on the approval of the study protocol must also be provided in the manuscript.
